# Biomarkers of osteoarthritis: translating information from in vitro culture systems to human patients

**DOI:** 10.1186/1471-2164-15-S2-O16

**Published:** 2014-04-02

**Authors:** Ali Mobasheri

**Affiliations:** 1 Faculty of Medicine and Health Sciences, The University of Nottingham, Sutton Bonington Campus, Sutton Bonington, LE12 5RD, UK; 2 D-BOARD European Consortium for Biomarker Discovery, Faculty of Medicine and Health Sciences, The University of Nottingham, Nottingham, NG7 2RD, UK; 3 Arthritis Research UK Centres for Pain, Sport, Exercise and Osteoarthritis, The University of Nottingham, Nottingham, NG7 2RD, UK; 4 Medical Research Council and Arthritis Research UK Centre for Musculoskeletal Ageing Research, The University of Nottingham, Nottingham, NG7 2UH, UK; 5 Schools of Life Sciences and Pharmacy, University of Bradford, Bradford BD7 1DP, UK; 6 Center of Excellence in Genomic Medicine Research (CEGMR), King AbdulAziz University, P.O. Box: 80216 Jeddah 21589, Saudi Arabia

## 

Biomarkers are anatomic, physiologic, biochemical, or molecular parameters that may be associated with the presence and severity of a specific disease. They are detectable by using a variety of methods, including physical examination, laboratory assays, and imaging and can be used as indicators of pharmacologic responses to therapeutic interventions. Despite the interest in biomarkers, there are currently no reliable, quantifiable and easily measured markers that provide an earlier diagnosis of arthritic diseases such as osteoarthritis (OA), especially during the asymptomatic and pre-radiographic stages of the disease. For example, many of the existing biomarkers of joint damage identify fragments of the extracellular matrix of cartilage (i.e. fragments of type II collagen, aggrecan and smaller proteoglycans). Identification of fragments of these molecules in urine or serum does not consistently correlate with radiographic changes and symptoms. Furthermore, biomarkers of type II collagen and aggrecan are not specific for the various stages of OA, and in some cases, may not even be specific for OA. Consequently, there are no reliable assays that can inform prognostic evaluations and monitor responses to therapeutic modalities. The lack of such biomarkers has also hampered the development of structure-modifying pharmacological agents for the treatment of OA. Current pharmacological management of this disease is dominated by analgesics, non-steroidal anti-inflammatory drugs (NSAIDs), slow-acting symptomatic drugs and intra-articular injection of hyaluronic acid (viscosupplementation) or corticosteroids. Analgesics and NSAIDs are commonly administrated to all patients, irrespective of the stage of the disease. However, clinical trials have shown that only 20 to 60 percent of patients actually respond to treatment. This means that many patients will not benefit from the drugs that are administered. Also, long-term use of analgesics and NSAIDs is associated with adverse reactions and gastro-intestinal side effects. Therefore, identification of more sensitive biomarkers of pre-radiographic joint tissue turnover have the capacity to reflect disease relevant biological activity and provide useful diagnostic and therapeutic information, enabling a more rational, targeted and individualized approach to disease management. The recent proliferation of post-genomic technologies has resulted in rapid growth and progress in biomarker research. Current research on OA biomarkers focuses on identification of biomarkers in urine, serum and synovial fluid using approaches including proteomics, metabolomics, lipidomics, advanced imaging techniques, genomics, epigenomics and transcriptmoics and bioinformatics. This presentation will discuss how these technologies are currently being applied to identify early biomarkers of joint inflammation and tissue damage using 2-D and 3-D culture systems and translating the information for the benefit of human patients. There is considerable interest in developing “combination biomarkers” that include biochemical, immunologic, genetic and imaging data. It is anticipated that “combination biomarkers” will have the diagnostic and prognostic power to predict responses to pharmaceutical agents and non-pharmacological therapies and facilitate patient sub-classification/stratification strategies to increase homogeneity in study and treatment groups. However, the availability of tools and reagents is a major bottleneck in the biomarker pipeline, which has consequences for the development of disease-modifying osteoarthritis drugs (DMOADs).

